# Crystal structure of 4-(4-meth­oxy­phen­yl)-7,7-dimethyl-2-methyl­amino-3-nitro-7,8-di­hydro-4*H*-chromen-5(6*H*)-one

**DOI:** 10.1107/S160053681401589X

**Published:** 2014-08-01

**Authors:** S. Antony Inglebert, Jayabal Kamalraja, K. Sethusankar, Paramasivam T. Perumal

**Affiliations:** a Sri Ram Engineering College, Chennai 602 024, India; b Organic Chemistry Division, CSIR–Central Leather Research Institute, Chennai 600 020, India; c Department of Physics, RKM Vivekananda College (Autonomous), Chennai 600 004, India

**Keywords:** crystal structure, chromene, intra­molecular hydrogen bonding, C—H⋯π inter­actions

## Abstract

In the title compound, C_19_H_22_N_2_O_5_, the six-membered carbocyclic ring of the chromene moiety adopts an envelope conformation with the dimethyl-substituted C atom as the flap. The pyran ring has a flat-boat conformation. The meth­oxy­phenyl ring is orthogonal to the mean plane of the chromene moiety, with a dihedral angle of 89.97 (8)°. The amine N atom deviates from the chromene mean plane by 0.1897 (16) Å. The methyl­amine and the nitro group are involved in an intra­molecular N—H⋯O hydrogen bond which generates an *S*(6) ring motif. They are slightly twisted out of the plane of the chromene moiety with torsion angles of C—N—C—O(pyran) = 2.2 (3)° and O(nitro)—N—C—C = −5.6 (2)°. In the crystal, there are only C—H⋯π inter­actions present, forming inversion-related dimers.

## Related literature   

For the biological and pharmacological properties of chromenes and their derivatives, see: Shah *et al.* (2013 ▶).For related structures, see: Narayanan *et al.* (2013 ▶); Inglebert *et al.* (2014 ▶).
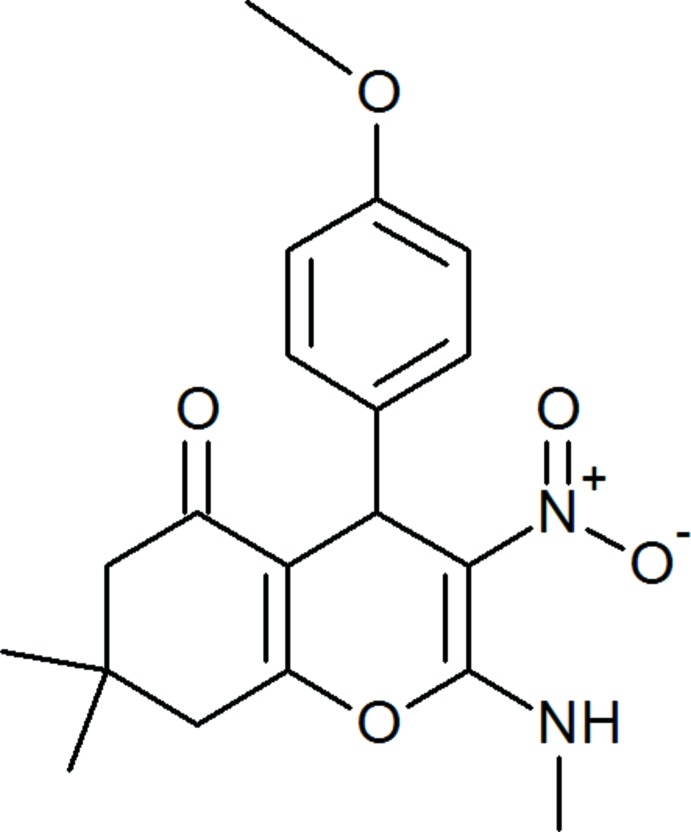


## Experimental   

### Crystal data  

C_19_H_22_N_2_O_5_
*M* *_r_* = 358.39
Monoclinic, 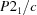

*a* = 9.6793 (7) Å
*b* = 16.3059 (12) Å
*c* = 11.9205 (8) Å
β = 106.128 (2)°
*V* = 1807.4 (2) Å^3^
*Z* = 4
Mo *K*α radiation
μ = 0.10 mm^−1^
*T* = 293 K
0.30 × 0.25 × 0.20 mm

### Data collection  

Bruker Kappa APEXII CCD diffractometer
Absorption correction: multi-scan (*SADABS*; Bruker, 2008 ▶) *T*_min_ = 0.972, *T*_max_ = 0.981
25137 measured reflections
3429 independent reflections
2369 reflections with *I* > 2σ(*I*)
*R*_int_ = 0.038

### Refinement  

*R*[*F* ^2^ > 2σ(*F* ^2^)] = 0.043
*wR*(*F* ^2^) = 0.140
*S* = 1.04
3429 reflections
239 parameters
H-atom parameters constrained
Δρ_max_ = 0.30 e Å^−3^
Δρ_min_ = −0.23 e Å^−3^

### 

Data collection: *APEX2* (Bruker, 2008 ▶); cell refinement: *SAINT* (Bruker, 2008 ▶); data reduction: *SAINT*; program(s) used to solve structure: *SHELXS97* (Sheldrick, 2008 ▶); program(s) used to refine structure: *SHELXL97* (Sheldrick, 2008 ▶); molecular graphics: *ORTEP-3 for Windows* (Farrugia, 2012 ▶); software used to prepare material for publication: *SHELXL97* and *PLATON* (Spek, 2009 ▶).

## Supplementary Material

Crystal structure: contains datablock(s) global, I. DOI: 10.1107/S160053681401589X/su2750sup1.cif

Structure factors: contains datablock(s) I. DOI: 10.1107/S160053681401589X/su2750Isup2.hkl

Supporting information file. DOI: 10.1107/S160053681401589X/su2750Isup3.cml

**Fig. 1.**
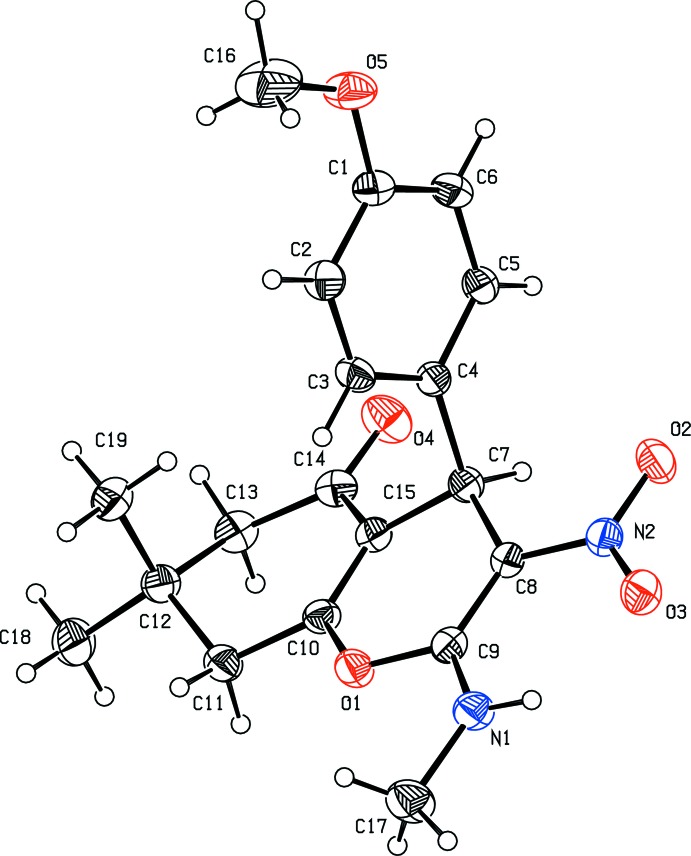
A view of the molecular structure of the title molecule, with atom labelling. Displacement ellipsoids are drawn at the 30% probability level.

. DOI: 10.1107/S160053681401589X/su2750fig1.tif
A view of the mol­ecular structure of the title mol­ecule, with atom labelling. Displacement ellipsoids are drawn at the 30% probability level.

CCDC reference: 1012691

Additional supporting information:  crystallographic information; 3D view; checkCIF report

## Figures and Tables

**Table 1:** Hydrogen-bond geometry (Å, °) *Cg*2 is the centroid of the C1–C6 ring.

*D*—H⋯*A*	*D*—H	H⋯*A*	*D*⋯*A*	*D*—H⋯*A*
C17—H17*B*⋯*Cg*2^i^	0.96	2.78	3.652	142
N1—H1⋯O3	0.86	1.98	2.602 (2)	128

Symmetry code: (i) 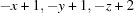.

## supplementary crystallographic information

### S1. Comment

Amino chromenes and their derivatives are an important class of heterocyclic compounds having significant biological activities. During the last decade, such compounds have shown interesting pharmacological properties including antimicrobial, mutagenicity, sex hormone, cancer therapy, and central nervous system activities (Shah *et al.*, 2013).

The title compound, Fig. 1, consists of a chromene unit attached to a methoxyphenyl ring, a nitro group, a dimethyl, a methyl-amine group and an oxygen atom. The mean plane of the chromene unit (O1/C7–C15) makes a dihedral angle of 89.87 (8)° with the phenyl ring (C1–C6), which shows that they are perpendicular to each other. The mean plane of the chromene unit makes dihedral angles of 5.56 (11) and 5.46 (9)° with the nitro and methylamine groups, respectively.

The six membered carbocyclic ring (C10–C15) of the chromene moiety adopts an envelope conformation with puckering parameters of Puckering Amplitude (Q) = 0.458 (2) Å, θ = 58.2 (3)° & φ = 120.1 (3)°. Atom C12 deviates by 0.323 (2) Å from the mean plane passing through the rest of the ring atoms. The title compound exhibits structural similarities with the reported related structures (Narayanan *et al.*, 2013; Inglebert *et al.*, 2014).

The amine group nitrogen atoms N1 and N2, deviate by -0.1897 (16) and -0.1081 (17) Å from the mean plane of the chromene moiety. The methyl amine group attached to C9 is coplanar with the chromene moiety as indicated by the torsion angle C17–N1–C9–O1 = 2.2 (3)°. The nitro group is also coplanar to the chromene moiety, as indicated by the torsion angles C9–C8–N2–O3 = -0.2 (3)° and C7–C8–N2–O2 = -5.6 (2)°, respectively.

The molecular structure is characterized by an intramolecular N—H···O hydrogen bond, generates an *S(6)* ring motif (Table 1).

In the crystal, there are only C—H···π interactions present (Table 1).

### S2. Experimental

A solution of 4-methoxylbenzaldehyde (1.0 mmol), 5,5-dimethylcyclohexane-1,3-dione (1.0 mmol), NMSM (1.0 mmol), and piperidine (0.2 eq) in the presence of ethanol (2 ml) was stirred for 5 h. After the reaction was complete, as indicated by TLC, the product was filtered out and washed with 2 ml of ethanol to remove the excess base and other impurities. The resulting products were recrystallized from ethanol yielding crystal of the title compound.

### S3. Refinement

H atoms were placed in idealized positions and allowed to ride on the parent atoms: N-H = 0.86 Å, C—H = 0.93 - 0.97 Å with *U*_iso_(H)= 1.5*U*_eq_(C-methyl) and = 1.2U_eq_(N,C) for other H atoms.

### Crystal data

**Table:**

C_19_H_22_N_2_O_5_	*F*(000) = 760
*M**_r_* = 358.39	*D*_x_ = 1.317 Mg m^−^^3^
Monoclinic, *P*2_1_/*c*	Mo *K*α radiation, λ = 0.71073 Å
Hall symbol: -p 2ybc	Cell parameters from 3429 reflections
*a* = 9.6793 (7) Å	θ = 2.2–25.7°
*b* = 16.3059 (12) Å	µ = 0.10 mm^−^^1^
*c* = 11.9205 (8) Å	*T* = 293 K
β = 106.128 (2)°	Block, colourless
*V* = 1807.4 (2) Å^3^	0.30 × 0.25 × 0.20 mm
*Z* = 4	

### Data collection

**Table:**

Bruker Kappa APEXII CCD diffractometer	3429 independent reflections
Radiation source: fine-focus sealed tube	2369 reflections with *I* > 2σ(*I*)
Graphite monochromator	*R*_int_ = 0.038
ω and φ scan	θ_max_ = 25.7°, θ_min_ = 2.2°
Absorption correction: multi-scan (*SADABS*; Bruker, 2008)	*h* = −11→11
*T*_min_ = 0.972, *T*_max_ = 0.981	*k* = −19→19
25137 measured reflections	*l* = −14→14

### Refinement

**Table:**

Refinement on *F*^2^	Primary atom site location: structure-invariant direct methods
Least-squares matrix: full	Secondary atom site location: difference Fourier map
*R*[*F*^2^ > 2σ(*F*^2^)] = 0.043	Hydrogen site location: inferred from neighbouring sites
*wR*(*F*^2^) = 0.140	H-atom parameters constrained
*S* = 1.04	*w* = 1/[σ^2^(*F*_o_^2^) + (0.0743*P*)^2^ + 0.445*P*] where *P* = (*F*_o_^2^ + 2*F*_c_^2^)/3
3429 reflections	(Δ/σ)_max_ < 0.001
239 parameters	Δρ_max_ = 0.30 e Å^−^^3^
0 restraints	Δρ_min_ = −0.23 e Å^−^^3^

### Special details

**Table:**

Geometry. All e.s.d.'s (except the e.s.d. in the dihedral angle between two l.s. planes) are estimated using the full covariance matrix. The cell e.s.d.'s are taken into account individually in the estimation of e.s.d.'s in distances, angles and torsion angles; correlations between e.s.d.'s in cell parameters are only used when they are defined by crystal symmetry. An approximate (isotropic) treatment of cell e.s.d.'s is used for estimating e.s.d.'s involving l.s. planes.
Refinement. Refinement of *F*^2^ against ALL reflections. The weighted *R*-factor *wR* and goodness of fit *S* are based on *F*^2^, conventional *R*-factors *R* are based on *F*, with *F* set to zero for negative *F*^2^. The threshold expression of *F*^2^ > σ(*F*^2^) is used only for calculating *R*-factors(gt) *etc*. and is not relevant to the choice of reflections for refinement. *R*-factors based on *F*^2^ are statistically about twice as large as those based on *F*, and *R*- factors based on ALL data will be even larger.

### Fractional atomic coordinates and isotropic or equivalent isotropic displacement parameters (Å^2^)

**Table:**

	*x*	*y*	*z*	*U*_iso_*/*U*_eq_	
C1	0.3188 (2)	0.60966 (13)	0.57925 (17)	0.0418 (5)	
C2	0.3527 (2)	0.64520 (13)	0.68892 (17)	0.0438 (5)	
H2	0.4155	0.6895	0.7066	0.053*	
C3	0.2913 (2)	0.61357 (12)	0.77209 (16)	0.0404 (5)	
H3	0.3137	0.6376	0.8457	0.049*	
C4	0.19830 (18)	0.54764 (11)	0.74909 (15)	0.0338 (4)	
C5	0.1649 (2)	0.51385 (12)	0.63813 (16)	0.0397 (5)	
H5	0.1009	0.4701	0.6200	0.048*	
C6	0.2248 (2)	0.54378 (13)	0.55413 (17)	0.0450 (5)	
H6	0.2020	0.5198	0.4805	0.054*	
C7	0.1347 (2)	0.51384 (11)	0.84356 (15)	0.0364 (4)	
H7	0.0713	0.4678	0.8108	0.044*	
C8	0.24959 (19)	0.48391 (11)	0.94849 (16)	0.0351 (4)	
C9	0.29187 (19)	0.52757 (11)	1.05297 (16)	0.0370 (4)	
C10	0.09369 (19)	0.61784 (12)	0.98350 (15)	0.0369 (4)	
C11	0.0227 (2)	0.68872 (12)	1.02260 (17)	0.0437 (5)	
H11A	0.0955	0.7254	1.0686	0.052*	
H11B	−0.0348	0.6692	1.0721	0.052*	
C12	−0.0740 (2)	0.73612 (12)	0.91906 (17)	0.0420 (5)	
C13	−0.1698 (2)	0.67469 (13)	0.83584 (18)	0.0454 (5)	
H13A	−0.2407	0.6538	0.8723	0.054*	
H13B	−0.2212	0.7035	0.7654	0.054*	
C14	−0.0921 (2)	0.60304 (12)	0.80176 (17)	0.0408 (5)	
C15	0.04735 (19)	0.57886 (11)	0.88193 (15)	0.0349 (4)	
C16	0.4646 (3)	0.7040 (2)	0.5103 (3)	0.0911 (10)	
H16A	0.4193	0.7489	0.5382	0.137*	
H16B	0.4845	0.7193	0.4386	0.137*	
H16C	0.5529	0.6903	0.5675	0.137*	
C17	0.4303 (3)	0.55740 (15)	1.25477 (19)	0.0601 (6)	
H17A	0.4608	0.6116	1.2410	0.090*	
H17B	0.5060	0.5305	1.3124	0.090*	
H17C	0.3463	0.5610	1.2824	0.090*	
C18	−0.1656 (3)	0.79636 (15)	0.9648 (2)	0.0601 (6)	
H18A	−0.2291	0.8249	0.9004	0.090*	
H18B	−0.1043	0.8352	1.0158	0.090*	
H18C	−0.2211	0.7670	1.0071	0.090*	
C19	0.0178 (2)	0.78296 (13)	0.85468 (19)	0.0513 (5)	
H19A	−0.0437	0.8135	0.7914	0.077*	
H19B	0.0739	0.7448	0.8244	0.077*	
H19C	0.0807	0.8199	0.9078	0.077*	
N1	0.39661 (17)	0.51069 (10)	1.14645 (13)	0.0440 (4)	
H1	0.4495	0.4688	1.1436	0.053*	
N2	0.32201 (17)	0.41331 (10)	0.93523 (14)	0.0408 (4)	
O1	0.21955 (14)	0.59653 (8)	1.06663 (11)	0.0443 (4)	
O2	0.28171 (16)	0.37471 (9)	0.84138 (13)	0.0546 (4)	
O3	0.42684 (15)	0.38750 (9)	1.01655 (13)	0.0517 (4)	
O4	−0.14344 (16)	0.56445 (10)	0.71241 (13)	0.0605 (4)	
O5	0.37227 (17)	0.63553 (10)	0.49054 (13)	0.0606 (5)	

### Atomic displacement parameters (Å^2^)

**Table:**

	*U*^11^	*U*^22^	*U*^33^	*U*^12^	*U*^13^	*U*^23^
C1	0.0398 (10)	0.0504 (12)	0.0366 (11)	0.0007 (9)	0.0128 (8)	0.0035 (9)
C2	0.0412 (11)	0.0462 (12)	0.0436 (12)	−0.0084 (9)	0.0110 (9)	−0.0018 (9)
C3	0.0432 (11)	0.0445 (11)	0.0322 (10)	−0.0045 (9)	0.0081 (8)	−0.0080 (8)
C4	0.0346 (9)	0.0337 (10)	0.0323 (10)	0.0019 (7)	0.0078 (7)	−0.0001 (8)
C5	0.0441 (11)	0.0368 (10)	0.0365 (11)	−0.0061 (8)	0.0084 (8)	−0.0053 (8)
C6	0.0526 (12)	0.0500 (12)	0.0319 (11)	−0.0018 (10)	0.0107 (9)	−0.0061 (9)
C7	0.0386 (10)	0.0352 (10)	0.0342 (10)	−0.0030 (8)	0.0082 (8)	−0.0022 (8)
C8	0.0391 (10)	0.0317 (10)	0.0351 (10)	0.0025 (8)	0.0112 (8)	0.0024 (8)
C9	0.0372 (10)	0.0366 (10)	0.0375 (11)	0.0015 (8)	0.0111 (8)	0.0043 (8)
C10	0.0376 (10)	0.0406 (11)	0.0317 (10)	0.0042 (8)	0.0085 (8)	0.0035 (8)
C11	0.0490 (11)	0.0441 (11)	0.0385 (11)	0.0079 (9)	0.0132 (9)	−0.0005 (9)
C12	0.0412 (10)	0.0443 (11)	0.0417 (11)	0.0070 (9)	0.0134 (9)	0.0036 (9)
C13	0.0341 (10)	0.0528 (12)	0.0486 (12)	0.0027 (9)	0.0103 (9)	0.0055 (10)
C14	0.0364 (10)	0.0482 (11)	0.0375 (11)	−0.0051 (9)	0.0097 (8)	0.0009 (9)
C15	0.0351 (9)	0.0360 (10)	0.0336 (10)	−0.0014 (8)	0.0094 (8)	0.0016 (8)
C16	0.091 (2)	0.115 (3)	0.0780 (19)	−0.0483 (19)	0.0415 (16)	0.0042 (18)
C17	0.0600 (14)	0.0704 (16)	0.0411 (12)	0.0102 (12)	−0.0009 (10)	−0.0052 (11)
C18	0.0615 (14)	0.0553 (14)	0.0684 (15)	0.0178 (11)	0.0262 (12)	0.0044 (12)
C19	0.0532 (12)	0.0467 (12)	0.0553 (13)	−0.0012 (10)	0.0174 (10)	0.0075 (10)
N1	0.0428 (9)	0.0486 (10)	0.0372 (9)	0.0080 (8)	0.0055 (7)	0.0017 (8)
N2	0.0439 (9)	0.0375 (9)	0.0428 (10)	0.0028 (7)	0.0149 (8)	0.0026 (8)
O1	0.0483 (8)	0.0448 (8)	0.0339 (7)	0.0104 (6)	0.0015 (6)	−0.0029 (6)
O2	0.0650 (10)	0.0459 (8)	0.0510 (9)	0.0057 (7)	0.0132 (7)	−0.0114 (7)
O3	0.0494 (8)	0.0493 (9)	0.0529 (9)	0.0145 (7)	0.0084 (7)	0.0039 (7)
O4	0.0483 (9)	0.0725 (11)	0.0514 (9)	0.0013 (8)	−0.0017 (7)	−0.0156 (8)
O5	0.0664 (10)	0.0765 (11)	0.0458 (9)	−0.0169 (8)	0.0269 (8)	0.0003 (8)

### Geometric parameters (Å, º)

**Table:**

C1—O5	1.367 (2)	C12—C18	1.522 (3)
C1—C2	1.384 (3)	C12—C13	1.529 (3)
C1—C6	1.386 (3)	C12—C19	1.530 (3)
C2—C3	1.389 (3)	C13—C14	1.505 (3)
C2—H2	0.9300	C13—H13A	0.9700
C3—C4	1.380 (3)	C13—H13B	0.9700
C3—H3	0.9300	C14—O4	1.218 (2)
C4—C5	1.386 (3)	C14—C15	1.474 (3)
C4—C7	1.528 (2)	C16—O5	1.408 (3)
C5—C6	1.378 (3)	C16—H16A	0.9600
C5—H5	0.9300	C16—H16B	0.9600
C6—H6	0.9300	C16—H16C	0.9600
C7—C15	1.504 (3)	C17—N1	1.456 (3)
C7—C8	1.505 (3)	C17—H17A	0.9600
C7—H7	0.9800	C17—H17B	0.9600
C8—N2	1.380 (2)	C17—H17C	0.9600
C8—C9	1.394 (3)	C18—H18A	0.9600
C9—N1	1.311 (2)	C18—H18B	0.9600
C9—O1	1.358 (2)	C18—H18C	0.9600
C10—C15	1.330 (3)	C19—H19A	0.9600
C10—O1	1.384 (2)	C19—H19B	0.9600
C10—C11	1.485 (3)	C19—H19C	0.9600
C11—C12	1.534 (3)	N1—H1	0.8600
C11—H11A	0.9700	N2—O2	1.248 (2)
C11—H11B	0.9700	N2—O3	1.265 (2)
			
O5—C1—C2	124.08 (18)	C14—C13—C12	115.15 (16)
O5—C1—C6	116.05 (17)	C14—C13—H13A	108.5
C2—C1—C6	119.87 (18)	C12—C13—H13A	108.5
C1—C2—C3	118.87 (18)	C14—C13—H13B	108.5
C1—C2—H2	120.6	C12—C13—H13B	108.5
C3—C2—H2	120.6	H13A—C13—H13B	107.5
C4—C3—C2	122.16 (17)	O4—C14—C15	120.41 (19)
C4—C3—H3	118.9	O4—C14—C13	121.60 (18)
C2—C3—H3	118.9	C15—C14—C13	117.96 (17)
C3—C4—C5	117.75 (17)	C10—C15—C14	118.59 (17)
C3—C4—C7	120.57 (16)	C10—C15—C7	122.52 (16)
C5—C4—C7	121.68 (16)	C14—C15—C7	118.83 (16)
C6—C5—C4	121.28 (18)	O5—C16—H16A	109.5
C6—C5—H5	119.4	O5—C16—H16B	109.5
C4—C5—H5	119.4	H16A—C16—H16B	109.5
C5—C6—C1	120.07 (18)	O5—C16—H16C	109.5
C5—C6—H6	120.0	H16A—C16—H16C	109.5
C1—C6—H6	120.0	H16B—C16—H16C	109.5
C15—C7—C8	108.89 (15)	N1—C17—H17A	109.5
C15—C7—C4	110.20 (15)	N1—C17—H17B	109.5
C8—C7—C4	111.93 (15)	H17A—C17—H17B	109.5
C15—C7—H7	108.6	N1—C17—H17C	109.5
C8—C7—H7	108.6	H17A—C17—H17C	109.5
C4—C7—H7	108.6	H17B—C17—H17C	109.5
N2—C8—C9	119.75 (16)	C12—C18—H18A	109.5
N2—C8—C7	117.19 (16)	C12—C18—H18B	109.5
C9—C8—C7	122.87 (16)	H18A—C18—H18B	109.5
N1—C9—O1	112.01 (16)	C12—C18—H18C	109.5
N1—C9—C8	128.20 (17)	H18A—C18—H18C	109.5
O1—C9—C8	119.79 (16)	H18B—C18—H18C	109.5
C15—C10—O1	122.61 (17)	C12—C19—H19A	109.5
C15—C10—C11	126.11 (17)	C12—C19—H19B	109.5
O1—C10—C11	111.28 (15)	H19A—C19—H19B	109.5
C10—C11—C12	111.81 (16)	C12—C19—H19C	109.5
C10—C11—H11A	109.3	H19A—C19—H19C	109.5
C12—C11—H11A	109.3	H19B—C19—H19C	109.5
C10—C11—H11B	109.3	C9—N1—C17	125.02 (17)
C12—C11—H11B	109.3	C9—N1—H1	117.5
H11A—C11—H11B	107.9	C17—N1—H1	117.5
C18—C12—C13	110.27 (17)	O2—N2—O3	120.24 (16)
C18—C12—C19	109.56 (18)	O2—N2—C8	118.77 (16)
C13—C12—C19	109.51 (16)	O3—N2—C8	120.99 (16)
C18—C12—C11	108.80 (17)	C9—O1—C10	120.16 (14)
C13—C12—C11	108.45 (16)	C1—O5—C16	118.32 (18)
C19—C12—C11	110.24 (16)		
			
O5—C1—C2—C3	179.89 (18)	C19—C12—C13—C14	69.9 (2)
C6—C1—C2—C3	0.1 (3)	C11—C12—C13—C14	−50.4 (2)
C1—C2—C3—C4	0.3 (3)	C12—C13—C14—O4	−157.06 (19)
C2—C3—C4—C5	−1.1 (3)	C12—C13—C14—C15	24.8 (3)
C2—C3—C4—C7	179.10 (17)	O1—C10—C15—C14	176.63 (16)
C3—C4—C5—C6	1.4 (3)	C11—C10—C15—C14	−4.4 (3)
C7—C4—C5—C6	−178.82 (17)	O1—C10—C15—C7	−6.2 (3)
C4—C5—C6—C1	−0.9 (3)	C11—C10—C15—C7	172.76 (18)
O5—C1—C6—C5	−179.63 (18)	O4—C14—C15—C10	−174.04 (19)
C2—C1—C6—C5	0.1 (3)	C13—C14—C15—C10	4.1 (3)
C3—C4—C7—C15	60.8 (2)	O4—C14—C15—C7	8.7 (3)
C5—C4—C7—C15	−119.02 (18)	C13—C14—C15—C7	−173.13 (16)
C3—C4—C7—C8	−60.5 (2)	C8—C7—C15—C10	17.8 (2)
C5—C4—C7—C8	119.66 (19)	C4—C7—C15—C10	−105.4 (2)
C15—C7—C8—N2	167.42 (15)	C8—C7—C15—C14	−165.09 (16)
C4—C7—C8—N2	−70.5 (2)	C4—C7—C15—C14	71.8 (2)
C15—C7—C8—C9	−17.7 (2)	O1—C9—N1—C17	2.2 (3)
C4—C7—C8—C9	104.4 (2)	C8—C9—N1—C17	−177.0 (2)
N2—C8—C9—N1	0.0 (3)	C9—C8—N2—O2	179.39 (17)
C7—C8—C9—N1	−174.71 (18)	C7—C8—N2—O2	−5.6 (2)
N2—C8—C9—O1	−179.21 (16)	C9—C8—N2—O3	−0.2 (3)
C7—C8—C9—O1	6.1 (3)	C7—C8—N2—O3	174.84 (16)
C15—C10—C11—C12	−23.7 (3)	N1—C9—O1—C10	−171.21 (16)
O1—C10—C11—C12	155.32 (16)	C8—C9—O1—C10	8.1 (3)
C10—C11—C12—C18	168.69 (17)	C15—C10—O1—C9	−8.3 (3)
C10—C11—C12—C13	48.7 (2)	C11—C10—O1—C9	172.65 (16)
C10—C11—C12—C19	−71.2 (2)	C2—C1—O5—C16	−1.8 (3)
C18—C12—C13—C14	−169.46 (17)	C6—C1—O5—C16	178.0 (2)

### Hydrogen-bond geometry (Å, º)

Cg2 is the centroid of the C1–C6 ring.

**Table:**

*D*—H···*A*	*D*—H	H···*A*	*D*···*A*	*D*—H···*A*
C17—H17*B*···*Cg*2^i^	0.96	2.78	3.652	142
N1—H1···O3	0.86	1.98	2.602 (2)	128

Symmetry code: (i) −*x*+1, −*y*+1, −*z*+2.
