# Supplementary figures and images for: Crystal structure of 4-(4-meth­oxy­phen­yl)-7,7-dimethyl-2-methyl­amino-3-nitro-7,8-di­hydro-4H-chromen-5(6H)-one
Source: Acta Crystallogr Sect E Struct Rep Online. 2014 Aug 1;70(Pt 9):o901–2. doi: 10.1107/S160053681401589X (PMC4186140; doi:10.1107/S160053681401589X)

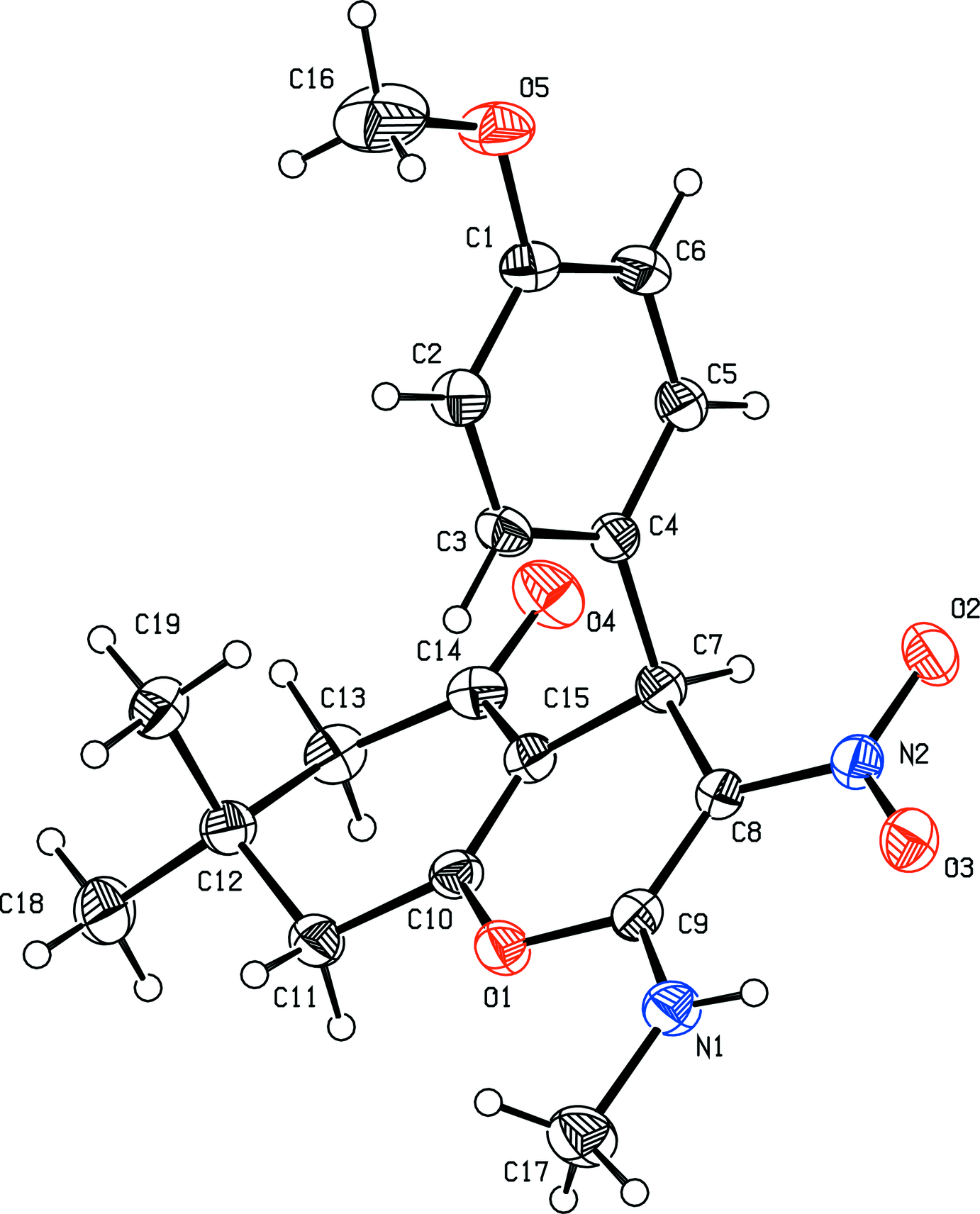

Supplement: Supplementary file 4 [file e-70-0o901-fig1.tif]
